# Porcine Mandibular Bone Marrow-Derived Mesenchymal Stem Cell (BMSC)-Derived Extracellular Vesicles Can Promote the Osteogenic Differentiation Capacity of Porcine Tibial-Derived BMSCs

**DOI:** 10.3390/pharmaceutics16020279

**Published:** 2024-02-16

**Authors:** Qun Zhao, Xing Zhang, You Li, Zhizhen He, Kang Qin, Eva Miriam Buhl, Ümit Mert, Klemens Horst, Frank Hildebrand, Elizabeth R. Balmayor, Johannes Greven

**Affiliations:** 1 Experimental Orthopedics and Trauma Surgery, University Hospital RWTH Aachen, Pauwelsstraße 30, 52074 Aachen, Germany; 2 Department of Orthopedics, Trauma and Reconstructive Surgery, University Hospital RWTH Aachen, Pauwelsstraße 30, 52074 Aachen, Germany; 3 Department of Shoulder and Elbow Surgery, Center for Orthopedic Surgery, The Third Affiliated Hospital of Southern Medical University, Guangzhou 510630, China; 4 Electron Microscopy Facility, Institute of Pathology and Medical Clinic II, University Hospital RWTH Aachen, 52074 Aachen, Germany

**Keywords:** mandible, BMSC, extracellular vesicles, bone regeneration, osteoblastic differentiation, osteogenesis

## Abstract

Objective: Existing research suggests that bone marrow-derived mesenchymal stem cells (BMSCs) may promote endogenous bone repair. This may be through the secretion of factors that stimulate repair processes or directly through differentiation into osteoblast-progenitor cells. However, the osteogenic potential of BMSCs varies among different tissue sources (e.g., mandibular versus long BMSCs). The main aim of this study was to investigate the difference in osteogenic differentiation capacity between mandibular BMSCs (mBMSCs) and tibial BMSCs (tBMSCs). Materials and Methods: Bioinformatics analysis of the GSE81430 dataset taken from the Gene Expression Omnibus (GEO) database was performed using GEO2R. BMSCs were isolated from mandibular and tibial bone marrow tissue samples. Healthy pigs (*n* = 3) (registered at the State Office for Nature, Environment, and Consumer Protection, North Rhine-Westphalia (LANUV) 81-02.04.2020.A215) were used for this purpose. Cell morphology and osteogenic differentiation were evaluated in mBMSCs and tBMSCs. The expression levels of toll-like receptor 4 (TLR4) and nuclear transcription factor κB (NF-κB) were analyzed using quantitative polymerase chain reaction (qPCR) and Western blot (WB), respectively. In addition, mBMSC-derived extracellular vesicles (mBMSC-EVs) were gained and used as osteogenic stimuli for tBMSCs. Cell morphology and osteogenic differentiation capacity were assessed after mBMSC-EV stimulation. Results: Bioinformatic analysis indicated that the difference in the activation of the TLR4/NF-κB pathway was more pronounced compared to all other examined genes. Specifically, this demonstrated significant downregulation, whereas only 5–7 upregulated genes displayed significant variances. The mBMSC group showed stronger osteogenic differentiation capacity compared to the tBMSC group, confirmed via ALP, ARS, and von Kossa staining. Furthermore, qPCR and WB analysis revealed a significant decrease in the expression of the TLR4/NF-κB pathway in the mBMSC group compared to the tBMSC group (TLR4 fold changes: mBMSCs vs. tBMSCs *p* < 0.05; NF-κB fold changes: mBMSCs vs. tBMSCs *p* < 0.05). The osteogenic differentiation capacity was enhanced, and qPCR and WB analysis revealed a significant decrease in the expression of TLR4 and NF-κB in the tBMSC group with mBMSC-EVs added compared to tBMSCs alone (TLR4 fold changes: *p* < 0.05; NF-κB fold changes: *p* < 0.05). Conclusion: Our results indicate that mBMSC-EVs can promote the osteogenic differentiation of tBMSCs in vitro. The results also provide insights into the osteogenic mechanism of mBMSCs via TLR4/NF-κB signaling pathway activation. This discovery promises a fresh perspective on the treatment of bone fractures or malunions, potentially offering a novel therapeutic method.

## 1. Introduction

Fractures cause a huge socioeconomic burden worldwide [1]. Deviations from the physiological process of fracture healing might cause persistent non-unions [2]. Currently, autografts are the gold standard treatment for fracture non-unions [3]. However, autografts are not always acceptable to patients because of prolonged postoperative pain and complications, such as infections and prolonged wound-healing hematomas, resulting in adverse reactions at the donor site [4,5]. In clinical practice, bone fracture non-union is still a hurdle for patients and doctors to overcome. Researchers have been working to find alternatives to promote osteogenic capacity at fracture sites in ways that are more acceptable to patients [6,7,8]. Many studies have investigated bone cells, including stem cells and osteoblasts, aiming to discover new approaches that promote bone healing.

Bone marrow mesenchymal stem cells (BMSCs) are non-hematopoietic, multipotent stem cells that can self-renew and differentiate into osteoblasts, adipocytes, and chondroblasts. This differentiation capacity plays a key role in tissue healing and regenerative medicine [9,10,11]. Not all BMSCs within the body exhibit the same osteogenic capacity after the in vitro induction of osteogenic differentiation. Studies involving rats [12,13] and humans [14] have shown that craniofacial BMSCs have a greater ability to proliferate and osteogenically differentiate than BMSCs derived from long bones [12,13] or iliac crests [14].

The osteogenic capacity of BMSCs is regulated by multiple factors, with extracellular vesicles (EVs) playing a significant role. Like many other cells, BMSCs release EVs. These EVs can be divided into two categories: ectosomes (also known as microvesicles) and exosomes. Ectosomes range in diameter from 50 nm to 1 μm, whereas the average diameter of exosomes is 100 nm, with a range of ~40 nm to 160 nm [15]. In the final stages of apoptosis, apoptotic cells can produce apoptotic EVs, known as ApoEVs. Exosomes contain various types of proteins, such as membrane proteins, cytosolic and nuclear proteins, extracellular matrix proteins, metabolites, and nucleic acids, including messenger RNA, miRNA, non-coding RNA species, and DNA [16,17,18,19]. The gene expression and signaling pathways of recipient cells can be affected when exosomes are taken up via phagocytosis, micropinocytosis, or the fusion of membranes, thereby causing functional changes in the cells [20]. Recently, exosomes have been studied as direct therapeutic agents or as vehicles for drug delivery [15].

In addition, studies have shown that stem cell-derived EVs can promote the osteogenic capacities of target cells in general and in diseases such as osteoporosis [21,22,23,24]. So far, only a few studies have investigated differences in the osteogenic capacities of BMSC-EVs derived from different tissue sources [12,13,14,25]. Based on previous studies, we hypothesize that BMSC-EVs have the capacity to promote osteogenesis. Little is known about BMSC-derived EV’s promotion of osteogenesis and the pathways or key proteins that may play important roles in these different traits.

Toll-like receptor 4 (TLR4) is an important protein involved in the regulation of osteogenic processes. It belongs to the family of pattern recognition receptors (PRRs). AlQranei et al. indicated that TLR4 may be involved in osteoclastogenesis by directly promoting osteoclast differentiation and inhibiting osteoblast differentiation [26]. A negative effect on bone metabolism may also be related to TLR-mediated inflammatory responses [27].

In this study, we focus on the osteogenic differentiation capacity of BMSCs obtained from different sources, namely tibia and mandibular bones, and investigate the potential of mBMSCs-EVs as biological carriers for enhancing osteogenesis in vitro. By comparing the bioinformatics analysis of the GSE81430 dataset with the results of our subsequent cell experiments, the possible reasons for the osteogenic differentiation capacity of BMSCs obtained from different sources were explored. Besides the known fact of differences in the osteogenic potential of BMSCs originating from different bones and the involvement of the LR4/NF-κB signaling pathway, it is still unknown how far these processes are influenced by mBMSC-EVs. Therefore, for the first time, this study will shed a light on the involvement of BMSC-EVs in osteogenic differentiation and their possible contribution to fractur healing.

## 2. Materials and Methods

### 2.1. Bioinformatics Analysis

#### 2.1.1. Gene Expression Omnibus (GEO) Database and Gene Microarray

The database investigations included microarray analysis of datasets comparing global gene expressions in BMSCs derived from the bone marrow of three pigs. The GEO Dataset (https://www.ncbi.nlm.nih.gov/geo/, accessed on 1 December 2021) search used the keyword “bone marrow mesenchymal stem cells”. Only Sus scrofa species were included. After checking the dataset’s titles and abstracts, the information of interest was separately evaluated in depth. Finally, only the GSE81430 dataset was chosen for further investigation [28].

Bioinformatics analyses of porcine mandibular-derived BMSC (mBMSC) and tibial-derived BMSC (tBMSC) expression matrixes were conducted. The methodologies for cell isolation (i.e., bone marrow from mandibles or tibiae) and analysis of the expression patterns (i.e., using high throughput analysis) were the same for all included studies.

The GSE81430 dataset is based on Platform GPL16493 ([PorGene-1_0-st] Porcine Gene 1.0 ST Array [transcript (gene) version]). Three mBMSC and tBMSC samples obtained from cultures at passage 0 were used. The National Center for Biotechnology Information (NCBI) GEO database (https://www.ncbi.nlm.nih.gov/geo, accessed on 1 December 2021) was used to generate the matrixes [28].

#### 2.1.2. Differentially Expressed Genes (DE Genes) and Target Genes

Initially, the expression matrix data of GSE81430 were normalized and preprocessed. The gene differential expressions of the mBMSC and tBMSC samples were then analyzed with the GEO2R comparison tool (https://www.ncbi.nlm.nih.gov/geo/geo2r/, accessed on 1 December 2021). The *p*-value was set at <0.05, and the fold change (FC) was set at >1.5 as the thresholds for identifying DE genes.

#### 2.1.3. Enrichment, Gene Ontology, and Pathway of the Target Genes

The platform GPL16493 performed functional annotations. David 2021 (https://david.ncifcrf.gov/home.jsp accessed on 1 December 2021) was used to convert gene IDs into gene names [29,30]. The STRING database (version 11.5) was used for functional annotation pathway enrichment analysis for the projected targets of the DE genes, Gene Ontology (GO), and Kyoto Encyclopedia of Genes and Genomes (KEGG) pathway analyses [31]. For further analysis, a *p*-value of <0.05 was considered statistically significant. The GO and KEGG enrichment plots were drawn using https://www.bioinformatics.com.cn (accessed on 1 December 2021.), an online platform for data analysis and visualization [32].

#### 2.1.4. Protein–Protein Interaction Network and Gene–Gene Network Construction

To further analyze functional relationships, protein–protein interactions (PPIs) and gene–gene networks (GGNs) were established using node pairs downloaded from the STRING database. The PPIs and GGNs were built using Cytoscape software (version 3.9.1) [33]. Only a cumulative interaction score greater than 0.4 was considered statistically significant. The degree of connectedness in the PPI network was then calculated using Cytoscape 3 software (GNU Lesser General Public License) according to the details of the node pairs [33]. Ranking was performed using cytoHubba (version 0.1—part of the Cytoscape software) according to the degree value of node pairs [34].

#### 2.1.5. Gene Set Enrichment Analysis for the TLR4/NF-κB Signaling Pathway

Gene set enrichment analysis (GSEA) was performed using GSEA software version 4.1.0 [35,36]. Before this analysis, a normalized expression matrix dataset (.gmt) and a phenotype label dataset (.cls) were created. Then, the TLR4/NF-κB signaling pathway analysis was performed using the TLR4/NF-κB gene set from MSigDB (http://software.broadinstitute.org/gsea/msigdb, accessed on 1 December 2021.) as input for the GSEA.

### 2.2. Verification of the Results via Quantitative Polymerase Chain Reaction and Western Blot

To further validate the findings, quantitative polymerase chain reaction (qPCR) and Western blot (WB) techniques were used.

#### Isolation and Cultivation of BMSCs

BMSCs were isolated from three porcine mandibles and tibias (ethical approval was registered at the State Office for Nature, Environment, and Consumer Protection, North Rhine-Westphalia [LANUV] 81-02.04.2020.A215) according to Lloyd et al.’s protocol [25,37]. Initially, bone marrow samples (*n* = 3) were aspirated from mandibular and tibial bone samples, and alpha-MEM Eagle L-Glutamine medium (Pan Biotech, Aidenbach, Germany, catalog number: P04-21051) was added. After centrifugation (1000× *g*, 3.5 min), each aspirate was cultured with alpha-MEM. The pellet was then re-suspended in alpha-MEM supplemented with 10% heat-inactivated fetal bovine serum (FBS) (Gibco, Grand Island, NY, USA, catalog number: 26140079) and 1% penicillin–streptomycin (Gibco, catalog number: 2257214); hereafter, this was used as the growth medium.

Next, the BMSCs were routinely cultured in tissue culture dishes (TPP, catalog number: 90076) containing growth medium and maintained at 37 °C in a humidified 5% CO_2_ atmosphere. Isolated cells were passaged until passage 3~5 for subsequent experiments. For osteogenic induction, BMSCs were maintained and further treated with an osteogenic induction medium containing 100 nM dexamethasone (Sigma-Aldrich, Schnelldorf, Germany, catalog number: 265005), 10 mM β-glycerophosphate (Sigma-Aldrich, catalog number: G9422), and 100 μM L-ascorbic acid-2-phosphate (Sigma-Aldrich, catalog number: A8960).

### 2.3. Isolation of Mandibular BMSC-Derived EVs

Prior to EV extraction, BMSCs were plated at an initial density of 1,000,000 cells per T75 flask. The BMSCs were cultured in a T75 flask until 80% confluency. The culture medium was replaced and supplemented with 13 mL of osteogenic induction medium and incubated for 14 consecutive days. Before the harvest day, the osteogenic induction medium was replaced with 13 mL of fresh 10% exosome-free FBS alpha-MEM and incubated for 24 h. To obtain a 10% exosome-free FBS alpha-MEM medium, FBS was ultracentrifuged for 16 h in ultracentrifugation tubes (Beckman Coulter, catalog number: 344058), following a previously reported procedure [38,39]. The osteogenic medium was sterilized and stored at 4 °C.

To harvest mBMSC-EVs, the cell supernatant was sequentially centrifuged at 300× *g* for 5 min, then at 2000× *g* for 15 min, and at 5000× *g* for 15 min. Thus, any cell debris was discarded. The supernatant was passed through 0.2 μm pore-size filters (VWR, Bruchsal, Germany, catalog number: 28145-501) before ultracentrifugation. Finally, mBMSC-EVs were pelleted via ultracentrifugation at 100,000× *g* for 90 min, resuspended in sterile PBS, and stored at −80 °C for further use. The temperature was maintained at 4 °C during the entire EV isolation process.

### 2.4. Characterization of Harvested mBMSC-EVs

#### 2.4.1. Nanoparticle Tracking Analysis Using NanoSight NS300

Nanoparticle tracking analysis (NTA) of the concentration of mBMSC-EVs and their sizes (mean diameter) was performed using NanoSight NS300 (NanoSight Technology 3.0 software, Malvern Panalytical, Malvern, UK). For all measurements, 1 mL of EV samples previously diluted with PBS (1:10) was injected into and passed through the measuring cuvette. The experiments were independently replicated three times.

#### 2.4.2. Transmission Electron Microscopy

Unfixed, isolated EVs were first treated with 0.1 M Hepes buffer and then absorbed for 10 min on 2 min glow-discharged formvar–carbon-coated nickel grids (Maxtaform, 200 mesh, Plano, Wetzlar, Germany). Samples were stained on grids by placing one drop of 0.5% uranyl acetate briefly in distilled water (Science Services GmbH, Munich, Germany). The samples were air dried and then examined using a transmission electron microscope (TEM) LEO 906 (Carl Zeiss, Oberkochen, Germany) at an acceleration voltage of 60 kV.

#### 2.4.3. CD9, CD63, and CD81 Fluorescein Labeling of EVs

The mBMSC-EVs were labeled using CD9 (BioLegend, London, UK, catalog number: 312105), CD63 (BioLegend, London, UK, catalog number: 353003), and CD81 (BioLegend, London, UK, catalog number: 349505). PKH67 (Sigma-Aldrich, Schnelldorf, Germany, catalog number: MINI67-1KT) was used as a positive control. Fluorescein-labeled EVs were then examined under a confocal laser scanning microscope (CLSM) (Zeiss, Jena, Germany) equipped with ZEN 2012 software (Zeiss, Zürich, Switzerland).

### 2.5. Cellular Uptake and Internalization of mBMSC-EVs

Wheat germ agglutinin (WGA) staining (Alexa Fluor™ 488 conjugate, Invitrogen™, Dreieich, Germany, catalog number: W11261) of the cell membranes was performed to analyze the cellular uptake and internalization of mBMSC-EVs. Before the experiments, BMSCs (50,000 cells/well) were seeded in specific confocal dishes (Thermo Scientific™, Dreieich, Germany, catalog number: 150680) and incubated for 24 h at 37 °C under humidified 5% CO_2_ conditions. Before testing, WGA-labeled EVs were rinsed three times with DPBS to avoid high background signals. The samples were centrifuged at 20,000× *g* for 90 min to remove excess WGA. Then, WGA-labeled EVs were added to alpha-MEM and incubated in confocal dishes for 1, 4, and 12 h at 37 °C, together with the tBMSCs. Finally, the number of stained EVs internalized into the cells was observed under a CLSM (Zeiss, Jena, Germany) equipped with ZEN 2012 software (Zeiss, Zürich, Switzerland).

### 2.6. Verification of BMSCs’ Gene Expression Using qPCR

The BMSCs (mBMSCs and tBMScs) at an initial density of 20,000 cells/well were maintained in the confocal culture dishes for 7 days. To further test the differentially expressed genes obtained from the bioinformatic analysis performed previously (namely, TLR4), qPCR was conducted on RNA samples extracted from isolated BMSCs. TRIzol™ Reagent (Invitrogen, Dreieich, Germany, catalog number: 15596018) was used to extract total RNA from all BMSC groups, following the manufacturer’s instructions (Primers used see Table 1). The groups were set up as follows: (i) tBMSC group = tibial BMSCs, (ii) mBMSC group = mandibular BMSCs, and (iii) tBMSC + EV group = tibial BMSCs with mandibular BMSC-derived EVs. Then, cDNA was synthesized using the High Capacity RNA-to-cDNA kit (Applied Biosystems, Zug, Switzerland, catalog number: 4387406), precisely following the provided instructions. Next, SYBR Green PCR Master Mix (Applied Biosystems, catalog number: 4309155) was used to perform qRT-PCRs on a CFX Opus 96 Real-Time PCR Instrument (Bio-Rad, Philadelphia, PA, USA) under the following settings: 95 °C for 3 min (initial denaturation), followed by 40 cycles of denaturation at 95 °C for 10 s. Annealing occurred at 55 °C for 30 s and elongation occurred at 72 °C for 1 min for each cycle, and for 5 min at 72 °C after the last cycle. Primers for forward and reverse directions were designed using Primer-BLAST (NCBI, Bethesda, MD, USA). Glyceraldehyde 3-phosphate dehydrogenase (GAPDH) was chosen as the housekeeping gene. All primers were commercially available from Eurofins.

A total of three cDNA samples of tBMSCs and mBMSCs, respectively, were tested, with duplicate tests performed for each sample. The 2ΔΔCt method was used to calculate the expression of each gene, and GAPDH was selected for normalization. Further normalization was performed by comparing the average ΔCt values for the tibial-derived samples to the average ΔCt values of the corresponding mandibular-derived samples. Gene expression was compared between sample sites (tibial to mandibular ratio) to calculate the fold-of-difference.

### 2.7. Verification of the Protein Expression Level Using Western Blot

The BMSCs (mBMSCs and tBMScs) at an initial density of 20,000 cells/well were maintained in the confocal culture dishes for 7 days. Gels containing 10% SDS-PAGE were used to separate equal amounts of total proteins from various BMSC samples at 80 V for 30 min and afterwards at 100 V for 1 h. A PVDF membrane (PerkinElmer, Rodgau, Germany, catalog number: 1620177) was used for protein transfer at 280 mA for 90 min. The cells were then blocked at room temperature for 2 h in the presence of 5% skimmed milk in a trimethylbenzene sulfonyl tetrazole buffer. This was gently shaken at 4 °C overnight with antibodies against TLR4 (1:1000, abcam, Berlin, Germany, catalog number: ab22048), NF-κB p65 Antibody (1:1000, Thermo Fisher Scientific, Dreieich, Germany, catalog number: PA5-27617), p-NF-κB p65 Antibody (1:1000, Thermo Fisher Scientific, Dreieich, Germany, catalog number: PA5-37718), and GAPDH antibody (1:1000, cell signaling technology, MA, USA, catalog number: 2118S). Then, the membranes were incubated with the corresponding secondary antibodies (Goat Anti-Mouse IgG H&L [HRP] [1:5000, Abcam, Berlin, Germany, catalog number: ab6789]; Goat Anti-Rabbit IgG [H + L] [1:5000, Proteintech, Planegg, Germany, catalog number: SA00001-2]) at room temperature for 30 min. As a final step, the immunoreactive protein bands were visualized using enhanced chemiluminescence reagents (ECL Substrate Kit, Abcam, Berlin, Germany, catalog number: ab133406) and examined on an LAS-3000 Imager (FUJIFILM, Tokyo, Japan).

### 2.8. Osteogenic Differentiation and Osteogenic Capacity Validation

The BMSCs (mBMSCs and tBMScs) at an initial density of 20,000 cells/well were maintained in the confocal culture dishes. For osteogenic differentiation, once the BMSC (mBMSCs and tBMScs) cultures attained 90% confluency, all BMSC groups were incubated with the osteogenic induction medium for up to 14 days. The culture media were exchanged every two days to steadily promote osteogenic differentiation and mineralization. After incubation for 14 days, alkaline phosphatase (ALP) staining (Abcam, Berlin, Germany, catalog number: ab242286), alizarin red staining (ARS) (Merck, Taufkirchen, Germany, catalog number: 3512879), and von Kossa staining (Sigma, Taufkirchen, Germany, catalog number: 100362) were performed to evaluate the osteogenic capacities of the various groups.

#### 2.8.1. ALP Staining

ALP staining was performed using an ALP kit (Abcam, Berlin, Germany, catalog number: ab242286) according to the manufacturer’s protocol. The various groups of BMSCs were rinsed with DPBS and fixed in 4% paraformaldehyde for 2 min at room temperature, followed by adding ALP staining solution in darkness for 30 min. Light microscopy was used to observe the images (Leica DMI 4000B, Wetzlar, Germany).

#### 2.8.2. ARS Staining

ARS staining (Merck, Taufkirchen, Germany, catalog number: 3512879) was performed on day 14 after osteoinduction according to the manufacturer’s protocol [40]. For ARS staining, BMSCs subjected to various treatments were washed using DPBS, fixed in 2% paraformaldehyde, and then stained with ARS working solution with orbital shaking at 100 rpm for 30min. The cells were washed in distilled water after staining was removed from the monolayers [41]. Light microscopy (Leica DMI 4000B, Wetzlar, Germany) was used to observe the stained mineralized nodules.

#### 2.8.3. Von Kossa Staining

Von Kossa staining was performed using the von Kossa kit (Sigma-Aldrich, Schnelldorf, Germany, catalog number: 1.00362.0001) and a previously described methodology [42]. Here, BMSCs in all pf the studied groups were rinsed with DPBS and fixed in 4% paraformaldehyde for 15 min, followed by staining with silver nitrate solution under UV exposure for 40 min. Then, sodium thiosulfate solution was added to indicate the minerals and matrixes of calcium deposits. The stained mineralized nodules were observed using a microscope (Leica DMI 4000B, Wetzlar, Germany).

### 2.9. Statistical Analysis

GraphPad Prism8.4.3 (GraphPad Software, San Diego, CA, USA) was used to perform the statistical analysis. Student’s *t*-test for pairwise comparisons and two-way ANOVA for comparisons among multiple groups were used to assess the significance between different groups. All data in our study were expressed as the mean and standard deviation (mean ± SD). Asterisks indicate significant differences: * *p*-value < 0.05, ** *p*-value < 0.01, and *** *p*-value < 0.001.

## 3. Results

### 3.1. Bioinformatics Analysis—DE Genes

**Figure 1:**
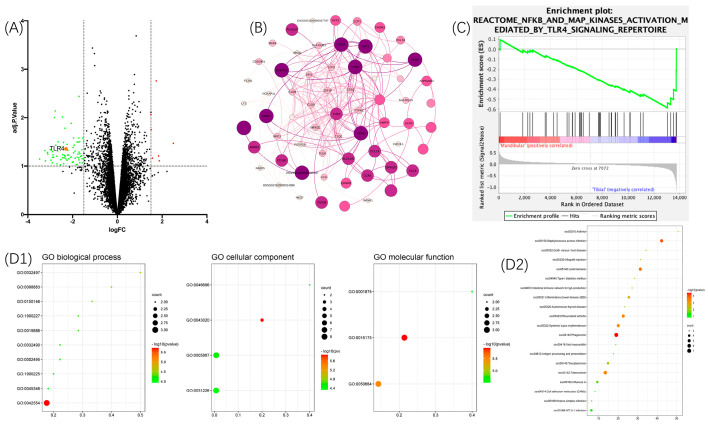
(**A**) The entire matrix volcano plots. Red labelled dots on the right indicated highly expressed genes and green dots on the left indicate lowly expressed genes. The highlighted red dot was TLR4. The fold change (FC) was set at > 1.5 as the thresholds for identifying DE genes. The horizontal dashed line in the graph represented adj. *p* value = 1, and the vertical dashed line represented the fold change (FC) equal to - 1.5 and 1.5. (**B**) Results of PPI network analysis. The top-ranked gene using cytoHubba was TLR4. (**C**) TLR4/NF-κB signaling pathway was significantly lower expressed in the mBMSC group. The *p* value calculated using GSEA software was *p* < 0.05. (**D1**) The top-ranked GO enrichment of BP, CC, and MF was the detection of lipopolysaccharide (GO: 0032497), lipopolysaccharide receptor complex (GO: 0046696), and lipopolysaccharide immune receptor activity (GO: 0001875). (**D2**) The top-ranked KEGG enrichment was ssc04145: Phagosome.

A volcano plot was created using GraphPad Prism8 (see Figure 1A) with the corresponding downloaded GEO matrix to obtain significantly downregulated or upregulated genes. The results showed that there were only four upregulated DE genes, whereas there were 28 downregulated DE genes. Consequently, this study focused on performing bioinformatics analysis for downregulated DE genes. The PPI network shown in Figure 1B indicates that the top-ranked gene is TLR4. Figure 1C shows the gene set enrichment analysis (GSEA): the TLR4/NF-κB signaling pathway was significantly lower expressed in the mBMSC group compared to the tBMSCs (*p*-value < 0.05). To connect the genes found to pathways and other proteins/genes, pathway analyses were performed using GO data sets and the KEGG. Figure 1(D1) shows that the top-ranked GO enrichment of the biological process (BP), the cellular component (CC), and the molecular function (MF) was the detection of lipopolysaccharides (GO: 0032497), lipopolysaccharide receptor complexes (GO: 0046696), and lipopolysaccharide immune receptor activity (GO: 0001875). The top-ranked KEGG enrichment was the ssc04145: Phagosome (Figure 1(D2)).

### 3.2. Characterization of mBMSC-EVs

**Figure 2:**
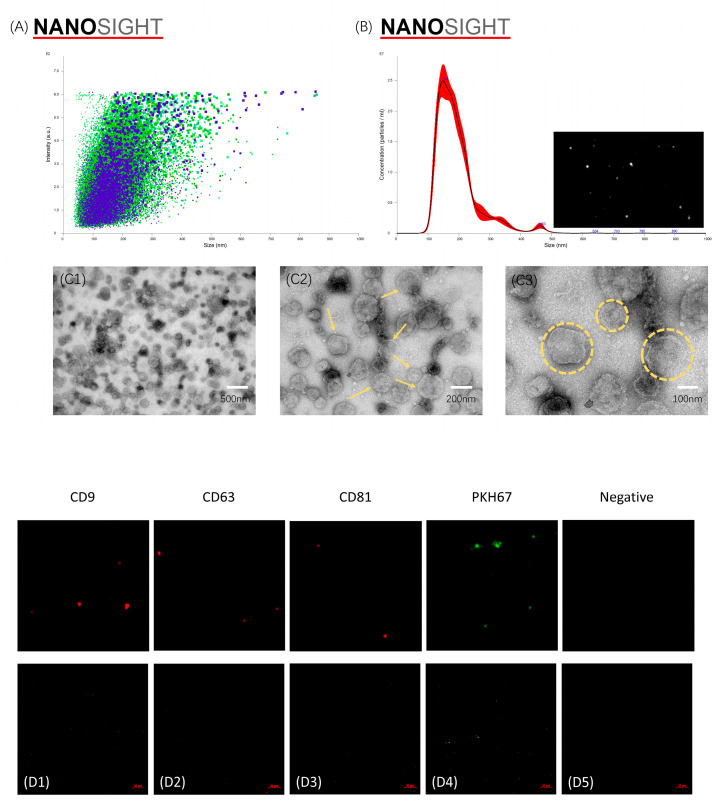
(**A**,**B**) Nanoparticle tracking analysis (NTA) of mBMSC-EVs. The different coloured dots represented the results of five repetitions of the measurement, with each colour representing one measurement. (**C1**–**C3**) Low- and high-magnification TEM images of BMSC-derived EVs. Yellow markings show that the mBMSC-EVs were spheroid-shaped with uniform distribution and a typical double-membrane nanostructure. (**D1**–**D5**) Low- and high-magnification confocal microscopy images of BMSC-derived EVs.

NanoSight analysis revealed that the obtained mBMSC-EVs concentration was (2.86 ± 0.22) × 10^9^ particles/mL. An average diameter of 194.7 ± 2.0 nm was observed for the EVs, ranging from 100 to 500 nm in diameter (Figure 2A,B). The TEM results showed a typical spheroidal double-membrane nanostructure, which supports the presence of exosomes (Figure 2 (C1–C3). The diameters of spherical vesicles determined using TEM ranged from 100–200 nm. Confocal microscopy images showed CD9, CD63, and CD81 positive staining of mBMSC-EVs (Figure 2(D1—D5)).

### 3.3. Verification of the Databank-Based Predicted Expression of TLR4 Using RT-qPCR and WB

**Figure 3:**
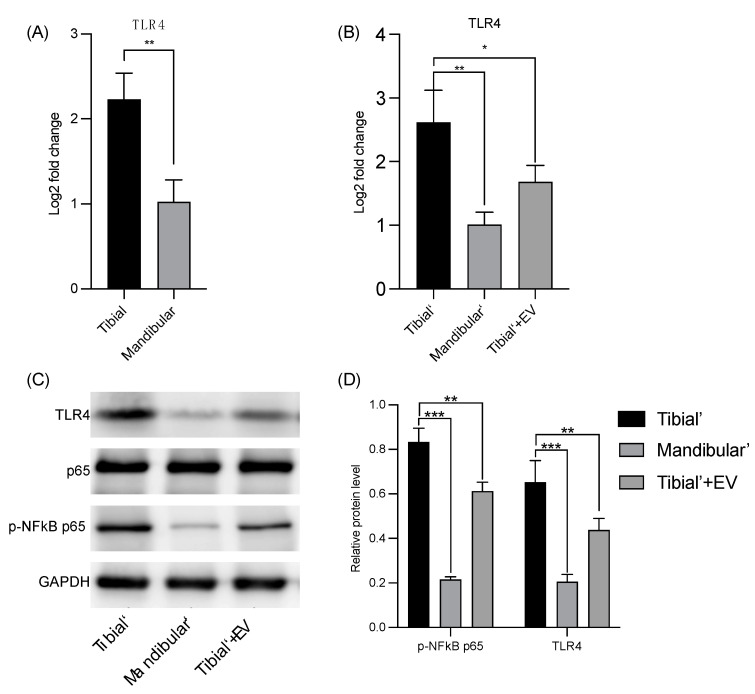
(**A**) TLR4 expression levels in mandibular and tibial BMSC groups using qPCR. (**B**) TLR4 expression levels using qPCR in various groups treated with osteogenic induction medium. (**C**) Representative WB image showing the protein levels of TLR4, p-NFκB p65, p65, and GAPDH in various groups. The TLR4 level was normalized using GAPDH (*n* = 3). The protein level of p-NFκB p65 was normalized using p65. (*n* = 3). (**D**) Western blot quantitative analysis. * indicated *p*-value < 0.05, ** indicated *p*-value < 0.01, *** indicated *p*-value < 0.001.

The results of the bioinformatic analysis were confirmed using RT-qPCR (Figure 3A). The RT-qPCR results show that TLR4 expression was significantly different between the mBMSC and tBMSC groups. 

To further verify the expression levels of TLR4 and NF-κB in each group, RT-qPCR and WB were performed. The mBMSC group had lower TLR4 expression levels compared to the tBMSC group (** *p*-value < 0.01, Figure 3B. Furthermore, the TLR4 expression of tBMSCs upon stimulation with mBMSC-EVs was significantly lower compared to the tBMSC group (* *p*-value < 0.05, Figure 3B). The results of the qualitative (Figure 3C) and quantitative WB analyses (Figure 3D) were consistent with those of the RT-qPCR of TLR4 and p-NF-κB p65 (phosphorylation of p65) and showed significantly different results for mBMSCs and tBMSCs. Both protein expression levels were lower for the mBMSC groups than for the tBMSC groups, *** *p*-value < 0.001. The expression levels of TLR4 and p-NF-κB p65 in the tBMSCs upon stimulation with the mBMSC-EV group were significantly lower compared to the tBMSC group, ** *p*-value < 0.01.

### 3.4. Cellular Uptake of mBMSC-EVs

**Figure 4:**
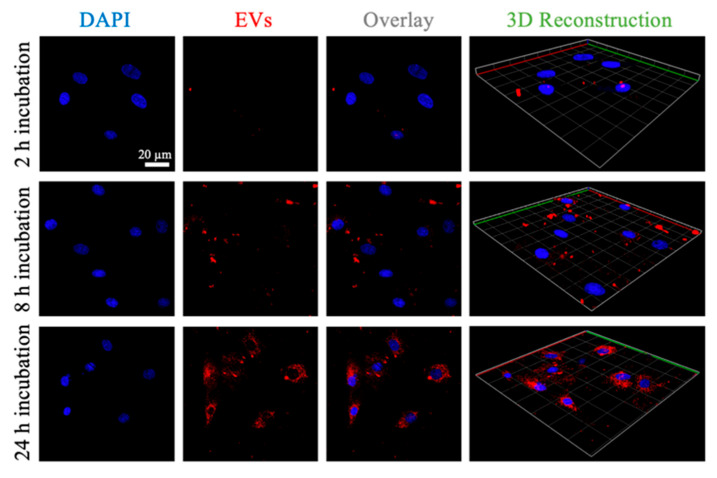
Confocal observation of mBMSC-EV cellular uptake at 0, 1, and 3 h. The nucleus was labeled using DAPI (blue). BMSC-derived EVs were labeled using wheat germ agglutinin (WGA). The 3D reconstruction images were observed using confocal microscopy.

To directly determine whether the mBMSC-EVs could be transferred into BMSCs, we visualized the cellular uptake processes using confocal laser scanning microscopy (CLSM). The labeled mBMSC-EVs were incubated together with BMSCs for different durations (1, 4, and 12 h). An increase in EV internalization by the BMSCs was observed with prolonged incubation times (Figure 4). EVs were distributed in the cytoplasm and around the cellular nucleus, as shown in the 2D and 3D reconstruction images (Figure 4).

### 3.5. Verification of the Osteogenic Capacity of BMSCs (ALP/ARS/Von Kossa)

**Figure 5:**
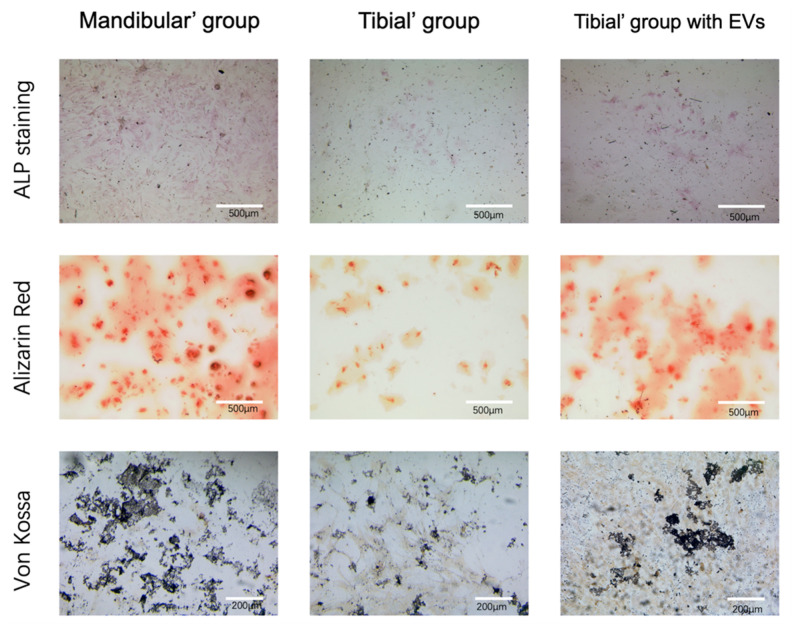
Representative images of osteogenic differentiation and osteogenic capacity validation for various groups using alkaline phosphatase staining, alizarin red staining, and von Kossa staining on day 14. Scale bar: 200 or 500 µm.

Next, we investigated the osteogenesis capacity of BMSCs in various groups. The BMSCs were cultured in an osteogenic differentiation medium. The mBMSC group cultured in osteogenic differentiation medium showed a stronger osteogenesis capacity than the tBMSC group cultured in the same medium. This was confirmed by the ALP, ARS, and von Kossa stainings, as shown in Figure 5. After mBMSC-EVs were added, the EV-stimulated tBMSC group showed stronger osteogenesis capacity compared to the nonstimulated tBMSC group (Figure 5), which indicated the osteogenic potential of the harvested mBMSC-EVs.

## 4. Discussion

Previous research has demonstrated that EVs generated and shed from stem cells can increase the osteogenic potential of osteoblasts [21,22,23,24]. Extracellular vehicles (EVs) are complex in composition, including proteins, nucleic acids (RNA and DNA), lipids, and metabolites. Key components such as proteins and nucleic acids play a key role in EV-mediated intercellular communication by participating in cell signaling, immune regulation, and gene expression regulation in receptor cells. To the best of our knowledge, a direct comparison of the osteogenic capacities of BMSCs from different sources following EV stimulation has not been reported before. Therefore, this study focused on the osteogenic differentiation capacities of mandibular and tibial BMSC sources and the potential of mandibular BMSC-secreted EVs as biological carriers for osteogenesis in vitro.

TLR4 has been investigated in bone healing and BMSCs in previous studies [43,44]. In this study, bioinformatics analysis demonstrated differential expression of TLR4 between mBMSCs and tBMSCs. Our results are consistent with other reports. Wang et al. inhibited the expression of TLR4 and found that this could promote the healing of bone defects in mice [43].

To ensure that mBMSC-EVs and cell interaction could be investigated without artificially induced artifacts (e.g., cell debris), the presence of EVs was first observed before performing cellular uptake assays. It was proven that isolated mBMSC-EVs were taken up by BMSCs. We also observed that the EVs were distributed in the cytoplasm and around the cellular nucleus. The mBMSC-EV uptake and distribution found in this study and their impact on cell homeostasis align with other studies [45,46,47].

Mineralized nodules in the mBMSC group indicated a significantly higher osteogenic capacity than those in the tBMSC group. The RT-qPCR results also showed that TLR4 expression was significantly higher in the tBMSC group, as further underlined by the histological results. The difference in TLR4 expression positively correlated with a difference in osteogenesis capacity. These findings agree with Guo et al. and Pei et al., who demonstrated the importance of the TLR4/NF-κB pathway in bone regeneration [48,49]. The addition of mBMSC-EVs to tBMSCs resulted in a significant increase in mineralized nodules, indicating increased osteogenic capacity. The RT-qPCR results showed that TLR4 expression decreased in the tBMSC group when EVs were added. A positive effect of mBMSC-EVs on bone healing has been shown in various studies [50,51]; however, none of them focused on the activation or inhibition of TLR4.

The expression of TLR4 depends on the activation of NF-κB, which is directly associated with the nuclear translocation of p65 [52]. Hence, stepwise phosphorylation of p65 promotes NF-κB activation. In this study, we determined p65 phosphorylation status using WB to determine the activation of the NF-κB pathway. Thus, it can be assumed that the higher the percentage of p65 phosphorylation, the stronger the activation of the NF-κB pathway [53]. The WB results showed that the phosphorylation of p65 decreased after mBMSC-EVs were added to the tBMSC culture.

Our findings show that TLR4 inhibits osteogenesis through TLR4-NF-κB signaling and mBMSC-EVs can downregulate TLR4 and p65 phosphorylation to promote osteogenesis in tBMSCs. These findings align with previous studies that showed that osteogenic capacity depends on NF-κB activity [44,54,55,56,57,58]. This might be partially due to the ability of EVs to carry and pass on specific microRNAs (miRNAs). Notably, miRNA-146a/b, expressed by BMSCs and encapsulated within their EVs, has been proven to exert inhibitory effects on TLR4 and its downstream targets IRAK1 and TRAF6, both integral components of the TLR4/NFκB pathway [59,60]. BMSC-derived EV-delivered miR-22-3p inhibits the MYC/PI3K/AKT pathway, promoting osteogenic differentiation via FTO repression [61]. BMSC-derived extracellular vesicles alleviate mouse osteoporosis via USP7-mediated YAP1 stability and Wnt/β-catenin pathway modulation [62]. Neural EGFL-Like 1-modified mesenchymal stem cell-derived extracellular vesicles enhance acellular bone regeneration through the miR-25-5p-SMAD2 signaling pathway [63]. The studies mentioned before suggest that exploring the impact of miRNA on osteogenesis is of great significance. We believe that mBMSC-EVs have significant potential for osteogenesis. In future studies, we plan to delve deeper into the mechanisms underlying mBMSC-EV-mediated osteogenesis, which could make mBMSC-EVs crucial in fracture repair within the field of tissue engineering. Donor-specific EVs could open up a field of personalized fracture healing treatments with no risk of graft rejection or other adverse effects like common clinic treatments [64,65].

## 5. Conclusions

Our study results showed that mBMSC-EVs promote osteogenesis. Although there was a strong similarity in the expression of the investigated BMSC genes from the two sources, there were also some significant expression differences. These expression differences in TLR4 may cause mBMSCs’ high osteogenic capacity toward tBMSCs, thereby promoting bone defects or fracture repairs. Finally, we were able to detect one of the key regulatory genes, TLR4, connected to the NF-κB pathway, which is directly regulated by EV uptake in the different bone sources. It highlighted the therapeutic potential of using mBMSC-EVs in regenerative medicine, specifically for repairing fractures or bone defects.

## Figures and Tables

**Table 1:** Primers used in this study.

Gene	Accession Number	Primer Sequences
*TLR4*	NM_001113039.2	Forward: 5′-CTGCCTTCACTACAGAGACTTCATTCC-3′
		Reverse: 5′-CACCACGACAATAACCTTCCGACTT-3′
*GAPDH*	NM_001206359.1	Forward: 5′-GTGAAGGTCGGAGTGAACGGATT-3′
		Reverse: 5′-ACCATGTAGTGGAGGTCAATGAAGG-3′

## Data Availability

The original contributions presented in the study are included in the article. Further inquiries should be directed to the corresponding authors.
